# A GDP-mannose-1-phosphate guanylyltransferase as a potential HIGS target against *Sclerotinia sclerotiorum*

**DOI:** 10.1371/journal.ppat.1013129

**Published:** 2025-05-02

**Authors:** Cheng Zhang, Yan Xu, Lin Li, Mingsong Wu, Zheyi Fang, Jinyi Tan, Jeffrey A. Rollins, Honghui Lin, Xinyi Huang, Shawn D. Mansfield, Xin Li, Yuelin Zhang

**Affiliations:** 1 Key Laboratory of Bio-resource and Eco-environment of Ministry of Education, College of Life Sciences, Sichuan University, Chengdu, China; 2 Michael Smith Laboratories, University of British Columbia, Vancouver, Canada; 3 Department of Botany, University of British Columbia, Vancouver, Canada; 4 Depertment of Plant Pathology, University of Florida, Gainesville, Florida, United States of America; Purdue University, UNITED STATES OF AMERICA

## Abstract

Sclerotinia stem rot is a devastating disease affecting vegetables and oil crops worldwide. It is caused by the necrotrophic ascomycete *Sclerotinia* (*S.*) *sclerotiorum.* Host-induced gene silencing (HIGS) has shown promise in disease control against insects and fungal pathogens, but effective HIGS target genes against *S. sclerotiorum* remain limited. In this study, we identified a GDP-mannose pyrophosphorylase (GMPP) SsMPG2 through forward genetic analysis. *Ssmpg2* mutants exhibit abnormal sclerotia and compound appressoria, along with defective cell wall integrity and attenuated virulence. Meanwhile, knocking out *SsMPG2* reduced the GMPP activity and glycosylation of proteins. In addition, SsMPG2 interacts with SsMPG1, which is essential in *S. sclerotiorum*. Downstream of the SsMPG1-SsMPG2 complex, *SsPMT4*, which encodes an O-mannosyltransferase, is also critical for compound appressoria formation and virulence. Notably, *MPG2* is essential for the virulence of several other fungal pathogens such as *Botrytis cinerea*, *Magnaporthe oryzae*, and *Fusarium graminearum*. Furthermore, expressing hairpin RNAs against *SsMPG1* and *SsMPG2* in *Nicotiana benthamiana* and *Arabidopsis thaliana* significantly reduced disease symptoms caused by *S. sclerotiorum*. Collectively, our findings demonstrate the critical roles of GMPP in the virulence of phytopathogenic fungi and suggest that *MPGs* are promising HlGS targets for controlling *S. sclerotiorum*.

## Author summary

Sclerotinia stem rot (SSR) is a devastating plant disease caused by *Sclerotinia* (*S.*) *sclerotiorum*. Oxalic acid (OA) is known to be a critical virulence factor for *S. sclerotiorum*. To search for OA-independent virulence factors, we screened for UV mutants in an OA-biosynthesis mutant background and found a mutant with reduced virulence. Next-generation sequencing analysis showed that its candidate gene encodes a GDP-mannose-1-phosphate guanylyltransferase (GMPP). Consistently, deletion of *SsMPG2* affects the GMPP activity and glycosylation of proteins. SsMPG1 and SsMPG2 are jointly involved in the synthesis of GDP-mannose, which in turn affects growth, cell wall integrity, compound appressoria formation, and virulence of *S. sclerotiorum*. Meanwhile, downstream of the SsMPG1-SsMPG2 complex, *SsPMT4*, encoding an O-mannosyltransferase, plays an important role in the growth and virulence of *S. sclerotiorum.* In addition, knocking out *MPG2* orthologs in multiple other phytopathogenic fungi also resulted in defects in growth, infection structure, and virulence, supporting its general importance in fungal virulence. Finally, host-induced gene silencing (HIGS) was successfully used to target *MPG1* and *MPG2* for controlling *S. sclerotiorum*. Our results provide new insights into fungal pathogenesis and identify *MPGs* as good gene targets to control *S. sclerotiorum*.

## 1. Introduction

Sclerotinia stem rot (SSR) is a destructive soilborne disease affecting many vegetable and oil crops worldwide. Several cash crops, including rapeseed, canola, soybean, and sunflower are particularly susceptible to SSR, which leads to wilting and maceration of host plant tissues [1,2]. In some regions of China, SSR can cause as much as 10%-20% reduction in rapeseed yield, resulting in significant economic losses [3]. The causal agent for SSR is *Sclerotinia* (*S.*) *sclerotiorum* (Lib.) de Bary [4]. As *S. sclerotiorum* forms sclerotia, the overwintering structures capable of surviving in soil for years, controlling the pathogen with chemical methods is challenging [5,6]. This highlights the urgent need for developing effective and sustainable strategies to manage *S. sclerotiorum* [7].

RNA interference (RNAi)-based technology can help enhance plant resistance against pathogens [8]. When double-stranded RNA (dsRNA) fragments of pests or pathogens’ origin are engineered into the host, they can lead to effective disease resistance through a process known as host-induced gene silencing (HIGS). HIGS designs involve expressing dsRNAs in the host to target and silence critical genes of pests or pathogens, thereby conferring resistance to the host [9,10]. For example, transgenic rice plants expressing hairpin RNAs targeting *MoAP1* showed enhanced resistance to *Magnaporthe* (*M.*) *oryzae*, inhibiting appressoria formation [11]. In addition, the *Verticillium dahliae* hydrophobin 1 (*VdH1*) is involved in fungal pathogenicity, and expressing an RNAi construct targeting *VdH1* in cotton enhanced resistance to *V. dahliae* [12].

HIGS has also been shown to be effective against *S. sclerotiorum* and *Botrytis cinerea*, with targeting genes encoding RAS signaling component SsGAP1 [13], the transcription module SsSnf5-SsHsf1-SsHsp70 [14], ABHYRDOLASE-3 [15], oxaloacetate acetylhydrolase SsOAH1 [16] and MAPK cascade component Ste50 [17]. However, the number of useful HIGS targets for the control of *S. sclerotiorum* remains low.

GDP-mannose pyrophosphorylase (GMPP) is a highly conserved enzyme, which can be found from bacteria to humans. It catalyzes the formation of GDP-mannose (GDP-Man) from mannose-1-phosphate (Man-1-P) and GDP in the cytosol [18]. Subsequently, GDP-Man is used to synthesize dolichol-phosphate-mannose (Dol-P-Man) in the endoplasmic reticulum (ER) [19]. Dol-P-Man acts as a sugar donor for protein glycosylation, making GMPP a rate-limiting enzyme in protein glycosylation [20,21]. The role of GMPP (also known as GDP-mannose-1-phosphate guanylyltransferase (MPG)) in protein glycosylation has been reported in various eukaryotes, including yeast, *Aspergillus fumigatus*, *Arabidopsis thaliana*, and *Homo sapiens* [22,23]. However, its function(s) in phytopathogenic fungi remain unclear.

In this study, we identified *SsMPG2* in *S. sclerotiorum* through a forward genetic screen designed to identify UV-induced mutants in the *Ssoah1* background that displays virulence defects [24,25]. *Ssmpg2* mutants cannot form normal sclerotia or compound appressoria, and exhibit defective cell walls and compromised virulence. Additionally, knocking out O-mannosyltransferase, *SsPMT4* (protein mannose transferase 4), downstream of GDP-Man in the biosynthesis pathway also led to defects in virulence. We further showed that the *SsMPG2* orthologs in *B. cinerea*, *M. oryzae*, and *Fusarium graminearum* also play similar roles in fungal development and virulence. Finally, we observed enhanced resistance against *S. sclerotiorum* in *Nicotiana benthamiana* and *Arabidopsis thaliana* plants expressing *SsMPG1* and *SsMPG2* dsRNAs, highlighting *SsMPG1 and SsMPG2* as effective HIGS targets for controlling SSR.

## 2. Results

### 2.1. UV mutant *S1093* carries a mutation in a gene encoding GDP-mannose pyrophosphorylase (GMPP) and exhibits reduced virulence, defective vegetative growth, and abnormal compound appressoria formation

**Fig 1:**
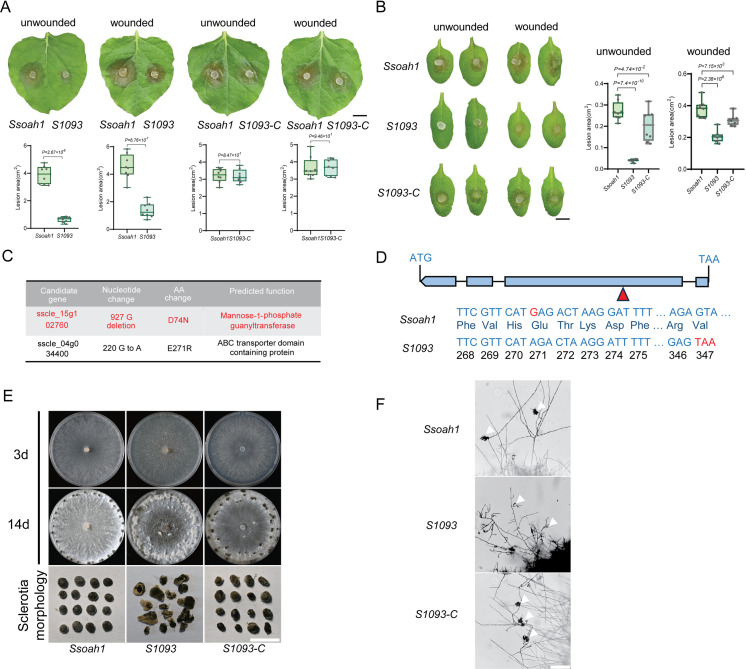
Phenotypic characterization of mutant *S1093* in the *S. sclerotiorum oah1* background. **(A)** Top: Virulence test of *Ssoah1*, *S1093*, and *S1093-C* on unwounded and wounded leaves of *N. benthamiana* at 24 hours post-inoculation (hpi). Bottom: Quantification of the lesion areas caused by the indicated *S. sclerotiorum* strains. The dots represent the values of lesion areas measured by ImageJ. The experiment was repeated twice with similar results. Bar = 0.5 cm. **(B)** Left: Virulence test of *Ssoah1*, *S1093*, and *S1093-C* on unwounded and wounded leaves of *A. thaliana* at 24 hpi. Right: Quantification of the lesion areas caused by the indicated *S. sclerotiorum* strains. The dots represent the values of lesion areas measured by ImageJ. The experiment was repeated twice with similar results. Bar = 0.2 cm. **(C)** List of candidate genes of *S1093* from NGS data analysis. Possible candidate genes are marked in red. **(D)** Diagram of genomic DNA differences between *Ssoah1* and *S1093* in *sscle_15g102760*. Base deletion and differential protein sequences are highlighted in red font. **(E)** Colony morphology and sclerotia morphology of *Ssoah1*, *S1093*, and *S1093-C* on PDA plates. The pictures were taken at 3 and 14 dpi, respectively. Bar = 0.5 cm. **(F)** Compound appressoria observation on glass slides of *Ssoah1*, *S1093*, and *S1093-C*. Representative photos were taken at 24 hpi. Bar = 100 μm. All statistical analyses were carried out by Student’s *t*-test.

Since oxalic acid (OA) plays a critical role in *S. sclerotiorum* disease progression, its presence can mask the contributions of other virulence factors [26]. Thus, to identify OA-independent virulence factors, we designed a forward genetic screen to search for mutants with reduced virulence in the *Ssoah1* OA-deficient background [24]. The lesion sizes on *N. benthamiana* leaves caused by the mutants and *Ssoah1* were compared. As shown in Fig 1A, the lesions caused by *S1093* were significantly smaller, and pre-inoculation wounding partly restored the lesion sizes to the *Ssoah1* level, suggesting a deficiency likely in host penetration. Similar results were observed on *Arabidopsis* leaves (Fig 1B).

To identify the causal mutations in *S1093*, we performed whole-genome next-generation sequencing (NGS) on its genomic DNA. Comparison with the reference genome identified two critical mutations in the coding regions of two genes, one with a single base-pair deletion, and the other with a point mutation (Fig 1C). The single base-pair deletion (927G deletion) in *sscle_15g102760* causes a predicted premature stop codon (Fig 1D). *sscle_15g102760* encodes the closest homolog of the mannose-1-phosphate guanylyltransferase (MPG2) in yeast and GDP-Mannose Pyrophosphorylase A (GMPPA) in humans, which are involved in the early steps of protein glycosylation [22,25]. As protein glycosylation is crucial to virulence in fungi [28], *sscle_15g102760* became the primary candidate gene for *S1093*. *sscle_15g102760* was subsequently renamed *SsMPG2*.

To test whether the mutation in *sscle_15g102760* is responsible for the *S1093* mutant phenotypes, we replaced the *SsMPG2*^927G deletion^ with the wild-type (WT) *SsMPG2* sequence in *S1093* via homologous recombination. The *S1093-C* strain grew at similar rates as *Ssoah1* on PDA media (S1 Fig). Regardless of unwounded or wounded, *Nicotiana benthamiana* and *Arabidopsis* leaves inoculated with the *S1093-C* strain exhibited similar lesion sizes as those inoculated with *Ssoah1* (Fig 1A and 1B).

Unlike *Ssoah1*, which forms black sclerotia at the edges of the PDA plates, *S1093* formed discolored sclerotia in the middle of the plates at day 14 (Fig 1E). For the *S1093-C* strain, sclerotia growth was similar to *Ssoah1* (S1A Fig). Since wounding facilitates the infection of *S1093*, we examined compound appressoria formation under the microscope. While mycelia of *Ssoah1* and *S1093-C* could form normal appressoria on glass slides, immature and malformed compound appressoria were observed in *S1093* (Fig 1F). Thus, the mutated *SsMPG2* in *S1093* is indeed the causal gene contributing to virulence, sclerotia development, and compound appressoria formation.

### 2.2. *SsMPG2* is involved in the development and virulence of *S. sclerotiorum*

**Fig 2:**
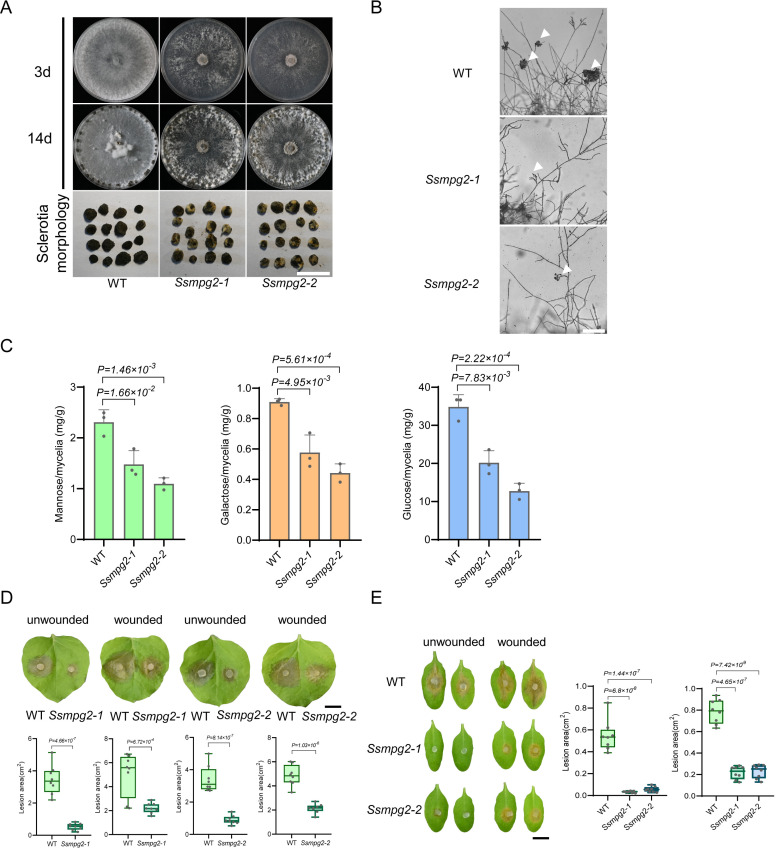
Knocking out *SsMPG2* in the wild type strain 1980 background leads to altered fungal development and virulence. **(A)** Colony and sclerotia morphology of wild type strain 1980 (WT) and two *Ssmpg2* deletion mutants on PDA plates. The pictures were taken at 3 and 14 dpi, respectively. Bar = 0.5 cm. **(B)** Compound appressoria observation on glass slides of WT and two *Ssmpg2* mutants. Representative photos were taken at 24 hpi. Bar = 100 μm. **(C)** Cell wall carbohydrate composition of WT and two *Ssmpg2* mutants. The ordinate represents the amount of sugar per milligram of hyphae, with at least three replicates per group. **(D)** Top: Virulence test of WT and two *Ssmpg2* mutants on unwounded and wounded leaves of *N. benthamiana* at 24 hpi. Bottom: Quantification of the lesion areas caused by the indicated *S. sclerotiorum* strains. The dots represent the values of lesion areas measured by ImageJ. The experiment was repeated twice with similar results. Bar = 0.5 cm. **(E)** Virulence test of WT and two *Ssmpg2* mutants on unwounded and wounded leaves of *A. thaliana* at 24 hpi. Quantification of the lesion areas caused by the indicated *S. sclerotiorum* strains is shown on the right. The dots represent the values of lesion areas measured by ImageJ. The experiment was repeated twice with similar results. Bar = 0.5 cm. All statistical analyses were carried out by Student’s *t*-test (**, P <= 0.01 *, P <= 0.05).

To further determine the biological function of *SsMPG2*, we constructed a knockout cassette targeting *SsMPG2* using homologous recombination in the WT strain 1980 background. Two independent deletion alleles were obtained and verified by PCR (S2A and S2B Fig). The presence of an amplified fragment within the *SsMPG2* gene in WT but not in *Ssmpg2–1* and *Ssmpg2–2*, along with the presence of the selectable marker gene *HPT* in only the two knockout mutants, confirmed the homozygosity of the deletion in the mutants. The two *Ssmpg2* mutants exhibited normal mycelial growth rates compared to WT on PDA media (S2C Fig). Moreover, the mutants formed discolored, deformed, immature sclerotia (Fig 2A). Meanwhile, malformed compound appressoria on glass slides were observed in the two *Ssmpg2* mutants (Fig 2B).

Since MPG2 contributes to cell wall integrity (CWI), which is essential for fungal pathogenicity [23,29,30], we examined the cell wall compositions of the two *Ssmpg2* mutants. As shown in Fig 2C, the content of mannose, galactose, and glucose was significantly lower in the *Ssmpg2* mutants than in WT. We further tested the virulence of the two mutants on *N. benthamiana* and *Arabidopsis* leaves. The lesions caused by their infection were much smaller compared to the WT, but were partially restored after pre-inoculation wounding (Fig 2D and 2E). These results were consistent with those observed in *S1093*, confirming that *SsMPG2* is the causal gene responsible for the *S1093* mutant phenotypes.

### 2.3. GMPP activity and protein glycosylation modifications are reduced in *Ssmpg2* mutants

**Fig 3:**
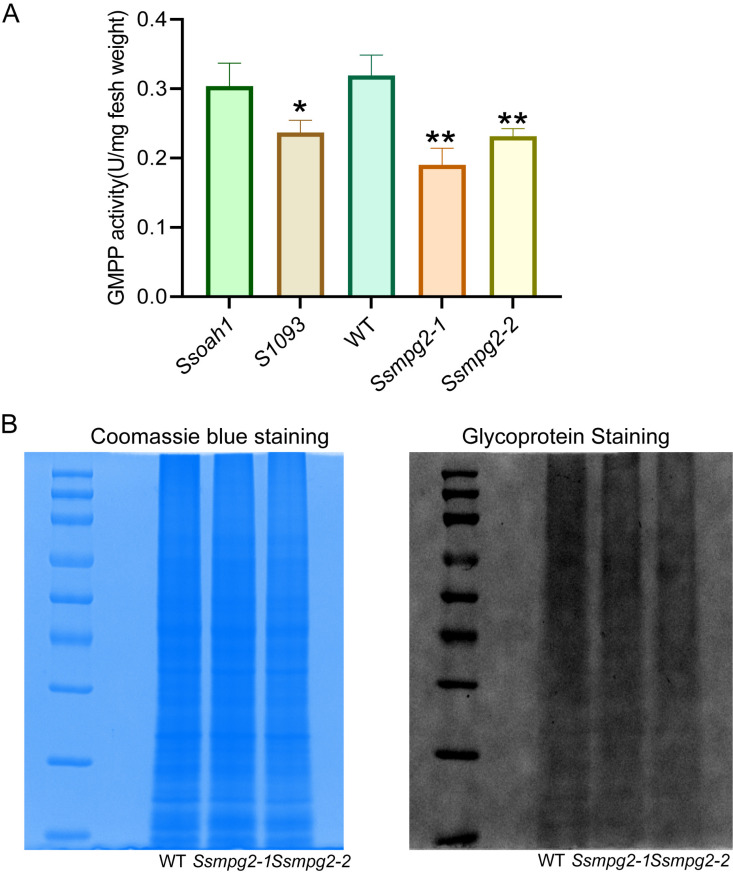
Functional analysis of *MPG2* in *S. sclerotiorum.* **(A)** GMPP activity assay in *Ssoah1*, *Ss1093*, WT, *Ssmpg2-1*, and *Ssmpg2-2*. The activity was assayed with 3-day-old mycelia grown on PDA, as measured by a GMPPase ELISA Kit. GMPP activity per milligram mycelium is shown. **(B)** The electrophoretic profiles of glycoproteins in the indicated genotypes. Proteins extracted from 3-day-old mycelia of WT, *Ssmpg2-1*, and *Ssmpg2-2* were separated by SDS-PAGE. Coomassie blue staining was performed for total protein detection, and glycosylated proteins were measured by a commercial Glycoprotein Staining Kit.

Phylogenetic analysis of *SsMPG2* from fungi, bacteria, *Arabidopsis*, and humans revealed that it belongs to a closely related GMPP clade. *SsMPG2* shares 30.31% sequence similarity with *SsMPG1* (S3A Fig). SsMPG2 has a two-amino-acid insertion in the highly conserved phosphate guanylyltransferase consensus motif (S3B Fig), similar to *Arabidopsis* KJC1 (KONJAC1) and KJC2 which lack GMPP enzymatic activity [30]. When the GMPP activity was measured in *S1093* and the two *Ssmpg2* deletion alleles by ELISA, significant reductions in GMPP activity were observed in all mutants compared to their respective controls (Figs 3A and S3C).

In eukaryotes, GDP-mannose is a key substrate for glycoprotein synthesis, and mannose is an essential monosaccharide for protein glycosylation [31]. To test whether the disrupted GDP-mannose synthesis in *Ssmpg2* mutants affects protein glycosylation, a glycoprotein staining assay was performed. Coomassie blue staining showed that the total protein amount for WT, *Ssmpg2–1*, and *Ssmpg2–2* used for the assay was almost identical. However, in the *Ssmpg2* mutants, the intensity of the glycoprotein bands was weaker compared to WT (Fig 3B). In conclusion, these data support a role of MPG2 in protein glycosylation in *S. sclerotiorum*.

### 2.4. SsMPG2 interacts with SsMPG1, and SsMPG1 is important for the survival and virulence of *S. sclerotiorum*

**Fig 4:**
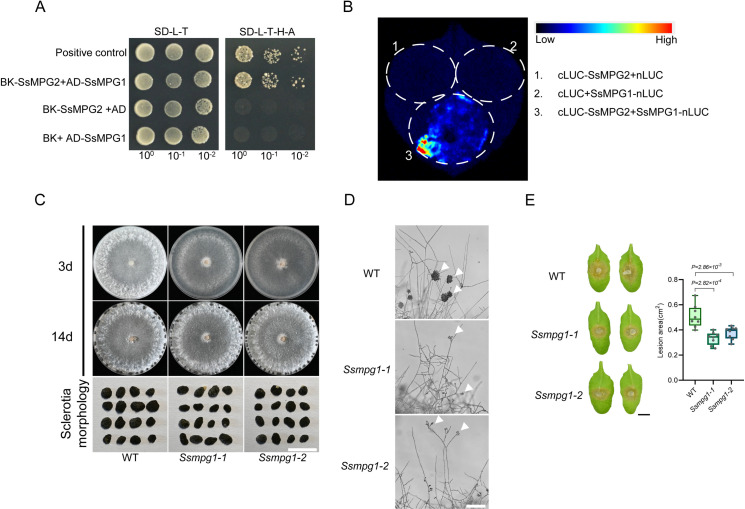
SsMPG2 interacts with SsMPG1 and SsMPG1 is essential for fungal development and virulence of *S. sclerotiorum.* **(A)** Yeast two-hybrid analysis of interactions between SsMPG2 and SsMPG1. Serial dilutions of yeast strains were prepared, and 10 μL of each dilution (OD_600_ = 10^0^, 10^1^, 10^2^) were spread onto synthetic dropout media lacking Leu and Trp (SD-L-T) or lacking Leu, Trp, His and Ade (SD-L-T-H-A). **(B)** Split luciferase complementation assay between SsMPG2 and SsMPG1 in *N. benthamiana*. Constructs expressing SsMPG2 fused with the C-terminus of the firefly luciferase fragment and SsMPG1 fused with the N-terminus of the firefly luciferase fragment were introduced into *Agrobacterium tumefaciens* strain GV3101, and the resulting bacteria were infiltrated into *N. benthamiana* leaves. After two days of incubation, the infiltrated leaves were infiltrated with 1 mM luciferin and the fluorescence signal was captured by a cooled charge coupled device (CCD) camera. The combinations of cLUC-SsMPG2 + nLUC and cLUC + SsMPG1-nLUC were used as the controls. **(C)** Colony and sclerotia morphology of WT and two *Ssmpg1* mutants on PDA plates. The pictures were taken at 3 and 14 dpi, respectively. Bar = 0.5 cm. **(D)** Compound appressoria observation on glass slides of WT and two *Ssmpg1* mutants. Representative photos were taken at 24 hpi. Bar = 100 μm. **(E)** Left: Virulence test of WT and two *Ssmpg1* mutants on the leaves of *A. thaliana* at 24 hpi. Right: Quantification of the lesion areas caused by the indicated *S. sclerotiorum* strains. The dots represent the values of lesion areas measured by ImageJ. The experiment was repeated twice with similar results. Bar = 0.5 cm. All statistical analyses were carried out by Student’s *t*-test.

In other organisms, such as *S. pombe*, MPG1 and MPG2 interact with each other and both are required for proper glycosylation [22]. Our yeast two-hybrid (Y2H) results supported the interaction of SsMPG2 with SsMPG1 (Fig 4A). Furthermore, the split luciferase complementation assay carried out in *N. benthamiana* confirmed that SsMPG2 can interact with SsMPG1 (Fig 4B).

To explore the role of *SsMPG1* in *S. sclerotiorum*, we attempted to knock out *SsMPG1*. However, we were unable to obtain a pure deletion mutant after multiple rounds of purification, indicating that a *Ssmpg1* null mutant might be lethal (S4A Fig). Two knockdown mutants, *Ssmpg1–1* and *Ssmpg1–2*, were however obtained, which had very low expression of *SsMPG1* (S4B Fig). These mutants showed similar mycelial growth as *Ssmpg2*, and they formed normal sclerotia (Figs 4C and S4C). Both *Ssmpg1* knockdown alleles developed deformed compound appressoria on glass slides (Fig 4D), and their virulence was attenuated on both *N. benthamiana* and *Arabidopsis* leaves (Figs 4E and S4D), suggesting that *SsMPG1* is essential for appressoria development and virulence.

### 2.5. *SsPMT4* contributes to *S. sclerotinia* growth, sclerotia development and virulence

**Fig 5:**
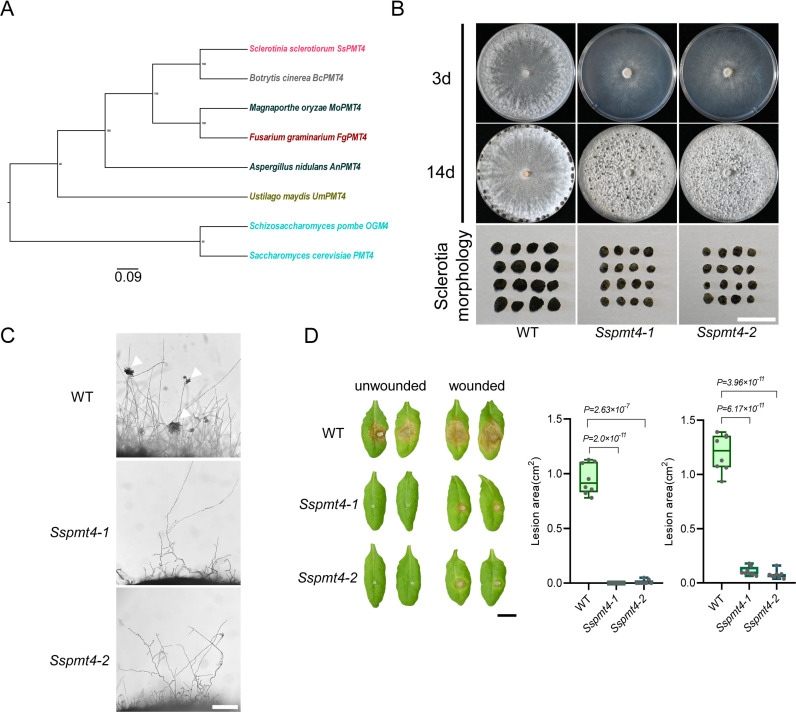
Functional analysis of *SsPMT4* in *S. sclerotiorum.* **(A)** Phylogenetic tree of PMT4 orthologs in representative fungi. The tree was built using Geneious, RaxmlGUI, and FigTree methods and evaluated by Bootstrap. The bootstrap values from 1000 replicates are labeled above the branches. The accession numbers of these proteins are APA05800.1 (SsPMT4), XP_024546940.1 (BcPMT4), XP_003713520.1 (MoPMT4), XP_011316298.1 (FgPMT4), ABH00990.1 (AnPMT4), XP_011392118.1 (UmPMT4), AJP39822.1 (OGM4), NP_596807.1 (PMT4). The scale bar is shown at the bottom. **(B)** Colony and sclerotia morphology of WT and two *Sspmt4* mutants on PDA plates. The pictures were taken at 3 and 14 dpi, respectively. Bar = 1 cm. **(C)** Compound appressoria observation on glass slides of WT and two *Sspmt4* mutants. Representative photos were taken at 24 hpi. Bar = 100 μm. **(D)** Top: Virulence test of WT and two *Sspmt4* mutants on the leaves of *A. thaliana* at 24 hpi. Bottom: Quantification of the lesion areas caused by the indicated *S. sclerotiorum* strains. The dots represent the values of lesion areas measured by ImageJ. The experiment was repeated twice with similar results. Bar = 0.5 cm. All statistical analyses were carried out by Student’s *t*-test for *P* values.

GDP-mannose is the precursor for all mannose residues in galactomannan, glycoproteins, and GPI anchors, which are essential for fungal cell wall synthesis and survival [32]. During protein glycosylation, O-mannosyltransferases (PMTs) in the endoplasmic reticulum act downstream of MPGs [33]. PMT1, PMT2, and PMT4 have been characterized in several plant pathogenic fungi, including *Ustilago maydis*, *Magnaporthe oryzae*, and *Botrytis cinerea*, and were shown to contribute to virulence [34–37]. Given the important roles of *PMT4* in various plant pathogenic fungi, we identified *sscle_01g005700* as the putative PMT4 ortholog in *S. sclerotiorum* using BLAST (Fig 5A).

To test the function of *SsPMT4* in *S. sclerotiorum*, *SsPMT4* was knocked out in the WT (S5A Fig). Two independent *Sspmt4* deletion mutants exhibited slower hyphal growth and formed smaller and discolored sclerotia compared to WT (Figs 5B and S5B). In addition, the two *Sspmt4* mutants failed to form compound appressoria on glass slides (Fig 5C). When we tested their virulence on both *N. benthamiana* and *Arabidopsis* leaves, lesions were completely absent in unwounded leaves, while smaller lesions appeared on pre-wounded leaves, particularly in *Arabidopsis* (Figs 5D and S5C). These results suggest that *SsPMT4* is crucial for compound appressoria formation, virulence, and sclerotia formation in *S. sclerotiorum*.

### 2.6. SsMPG2 orthologs play critical roles in virulence of multiple phytopathogenic fungi

**Fig 6:**
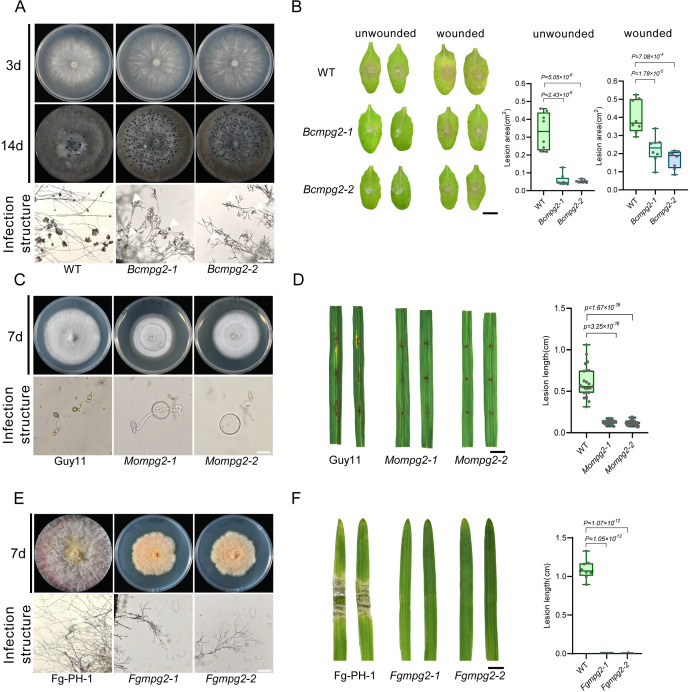
Knocking out *MPG2* in *B. cinerea*, *M. oryzae*, and *F. graminearum* affects fungal development and virulence. **(A)** Colony morphology of *B. cinerea* B05.10 (WT) strain and two independent *Bcmpg2* deletion mutants on PDA plates. The pictures were taken at 3 and 14 dpi, respectively. Bottom: Compound appressoria observation on glass slides of *B. cinerea* WT and *Bcmpg2* mutants. Representative photos were taken at 24 hpi. Bar = 100 μm. **(B)** Left: Virulence test of *B. cinerea* WT and two independent *Bcmpg2* deletion alleles on unwounded and wounded leaves of *A. thaliana* at 24 hpi. Right: Quantification of the lesion areas caused by the indicated *B. cinerea* strains. The dots represent the values of lesion areas measured by ImageJ. The experiment was repeated twice with similar results. Bar = 0.5 cm. **(C)** Colony morphology of *M. oryzae* WT strain Guy11 and two *Mompg2* mutants on Complete Medium (CM) plates. The pictures were taken at 8 dpi. Bottom: Appressoria observation on glass slides of *M. oryzae* WT and two *Mompg2* mutants. Representative photos were taken at 12 hpi. Bar = 10 μm. **(D)** Left: Virulence test of *M. oryzae* WT and two independent *Mompg2* deletion alleles on wounded leaves of rice at 7 dpi. Right: Quantification of the lesion areas caused by the indicated *M. oryzae* strains. The dots represent the values of lesion length measured by ImageJ. The experiment was repeated twice with similar results. Bar = 0.5 cm. **(E)** Colony morphology of *F. graminearum* strain PH-1 (WT) and two *Fgmpg2* deletion mutants on PDA plates. The pictures were taken at 7 dpi. Bottom: Infection cushions on glass slides of *F. graminearum* WT strain PH-1 and two *Fgmpg2* mutants. Representative photos were taken at 48 hpi. Bar = 100 μm. **(F)** Left: Virulence test of *F. graminearum* WT and two independent *Fgmpg2* deletion allele on wheat at 3 dpi. Right: Quantification of the lesion areas caused by the indicated *F. graminearum* strains. The dots represent the values of lesion length measured by ImageJ. The experiment was repeated twice with similar results. Bar = 0.5 cm. All statistical analyses were carried out by Student’s *t*-test for *P* values.

Having demonstrated the role of *MPG2* in *S. sclerotiorum*, we further tested whether the MPG2 orthologs play similar functions in other phytopathogenic fungi, including *B. cinerea*, *M. oryzae*, and *F. graminearum.* First, we obtained two independent *BcMPG2* deletion mutants, *Bcmpg2–1* and *Bcmpg2–2*. Similar to the *Ssmpg2* mutants, *Bcmpg2–1* and *Bcmpg2–2* exhibited abnormal vegetative growth on PDA plates, forming smaller and increased number of sclerotia at 14 days (Figs 6A and S6A). In addition, malformed compound appressoria on glass slides was observed in the *Bcmpg2* mutants (Fig 6A). In infection assays on *Arabidopsis* leaves, virulence of the *Bcmpg2* mutants was significantly attenuated compared to the *B. cinerea* WT strain B05.10, which was partially restored with pre-inoculation wounding (Fig 6B).

Next, we knocked out *MPG2* in *M. oryzae* and *F. graminearum* (S6B and S6C Fig). Both *Mompg2* and *Fgmpg2* mutants exhibited growth defects on plates, indicating that *MPG2* affects fungal growth and development (Fig 5C and 5E). As *MPG2* is necessary for compound appressoria formation in *S. sclerotiorum* and *B. cinerea*, we then examined whether infection structures were affected in *Mompg2* and *Fgmpg2* mutants. In *Mompg2* mutants, the appressoria from conidia were much larger than WT Guy11 (Fig 5C), which is likely due to the disability of cell wall to withstand the turgor pressure within the appressoria [38]. In *Fgmpg2* mutants, due to the severe inhibition of hyphal growth, the infection cushions were not observed (Fig 5E). Consistently, *Mompg2* and *Fgmpg2* mutants exhibited dramatically reduced virulence on their hosts (Fig 5D and 5F). Taken together, these results demonstrate that *MPG2* plays broad roles in vegetative growth and virulence in phytopathogenic fungi.

### 2.7. HIGS of *SsMPG* in *N. benthamiana* and *Arabidopsis* reduces *S. sclerotiorum* virulence

**Fig 7:**
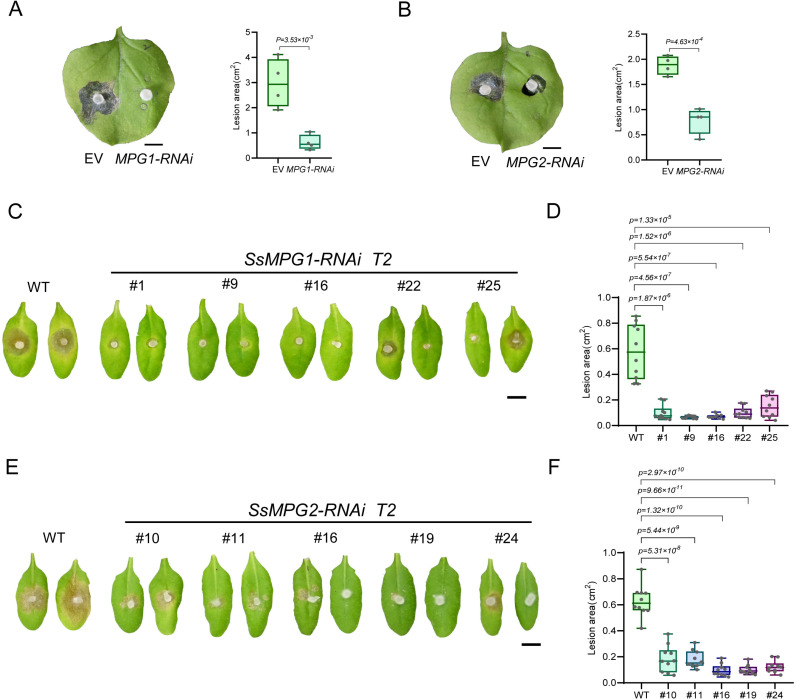
HIGS of *SsMPGs* reduces the virulence of wild type *S. sclerotiorum* in *N. benthamiana* and stable transgenic *A. thaliana.* **(A)** Virulence test of *S. sclerotiorum* on *N. benthamiana* leaves expressing EV (left) or *SsMPG1*-RNAi (right) constructs. The picture was taken at 48 hpi. Bar = 0.5 cm. **(B)** Virulence test of *S. sclerotiorum* on *N. benthamiana* leaves expressing EV (left) or *SsMPG2*-RNAi (right) constructs. The picture was taken at 48 hpi. Bar = 0.5 cm. **(C)** Virulence test of *S. sclerotiorum* on transgenic *A. thaliana* plants expressing pC1300-*SsMPG1*-RNAi-E9 constructs in T2 generation. Representative photos were taken at 48 hpi. Bar = 0.5 cm. **(D)** Quantification of the lesion areas caused by *S. sclerotiorum*. The dots represent the values of lesion areas measured by ImageJ. The experiment was repeated twice with similar results. **(E)** Virulence test of *S. sclerotiorum* on WT *A. thaliana* and transgenic *A. thaliana* plants expressing pC1300-*SsMPG2*-RNAi-E9 constructs in T2 generation. Representative photos were taken at 48 hpi. Bar = 0.5 cm. **(F)** Quantification of the lesion areas caused by *S. sclerotiorum* WT. The dots represent the values of lesion areas measured by ImageJ. The experiment was repeated twice with similar results. All statistical analyses were carried out by Student’s *t*-test for *P* values.

Given that *SsMPGs* are required for the virulence of *S. sclerotiorum*, we tested whether they can serve as HIGS targets for disease control. We selected a 422-bp DNA sequence from the third exon of *SsMPG1* and a 366-bp DNA sequence from the second exon of *SsMPG2* to generate dsRNA (S7 Fig). As shown in Fig 7A and 7B, the expression of *MPG1*-RNAi and *MPG2*-RNAi in *N. benthamiana* leaves significantly attenuated the virulence of *S. sclerotiorum*, and the effects of *MPG1*-RNAi appeared more obvious.

To test the effectiveness of HIGS in stable transgenic plants, the *MPG1*-RNAi and *MPG2*-RNAi constructs were independently transformed into *A. thaliana* WT plants. In T1 generation, 10 out of 33 *SsMPG1* RNAi transgenic lines exhibited smaller lesions compared to *A. thaliana* WT plants. In T2 generation, 5 of these lines (#1, #9, #16, #22, and #25) showed significantly enhanced resistance to *S. sclerotiorum* infection (Fig 7C and 7D). In parallel, 9 out of 31 T1 *SsMPG2* RNAi transgenic lines exhibited smaller lesions compared to *A. thaliana* WT plants. In T2 generation, 5 of these lines (#10, #11, #16, #19, and #24) showed significantly enhanced resistance to *S. sclerotiorum* infection (Fig 7E and 7F). These results suggest that *SsMPG1* and *SsMPG2* can be used as HIGS targets to control *S. sclerotiorum* infection.

## 3. Discussion

Protein glycosylation is a ubiquitous post-translational modification in eukaryotes [39]. GDP-mannose, synthesized by GDP-mannose pyrophosphorylase (GMPP/MPG) from GTP and mannose-1-phosphate, acts as a mannose donor for protein glycosylations [40]. Despite its importance, GMPP has not been well studied in phytopathogenic fungi. In our study, we found that in *S. sclerotiorum*, SsMPG1 and SsMPG2 play crucial roles in hyphal growth, compound appressoria formation, cell wall integrity, and virulence. Additionally, *SsMPG2* and *SsMPG1* can be used as HIGS targets to control Sclerotinia stem rot.

Phylogenetic analysis clearly showed that MPGs are highly conserved across eukaryotes. In humans, mutations in *GMPPA* or *GMPPB* cause congenital disorders of glycosylation and muscular dystrophies [41,42]. Mechanistically, GMPPA maintains GDP-mannose homeostasis by allosterically regulating GMPPB activity [27]. VTC1 (VITAMIN C DEFECTIVE 1) in *Arabidopsis* has been identified as a GMPP involved in L-ascorbic acid (AsA) synthesis [43,44], while KJC1 and KJC2 interact with VTC1 to stimulate GMPP activity, regulating plant growth and development by affecting AsA levels and glucomannan accumulation [30]. A GMPP homolog has also been characterized in *Schizosaccharomyces pombe* (MPG1), which is essential for maintaining cell wall integrity and glycosylation. The absence of *MPG1* affects septum structure and cell division [45]. MPG2, a homolog of MPG1, interacts and forms complexes with MPG1 to support glycosylation, and the overexpression of *MPG1* can partially compensate for the defects of *MPG2* deletion [22]. Repression of the GMPP-encoding *CaSRB1 (SRB1/PSA1* homolog) gene in *Candida albicans* has been shown to lead to a loss of viability and increased sensitivity to several antifungal agents and cell wall inhibitors [46]. Similarly, in *Aspergillus fumigatus*, the Af*srb1* gene is essential for cell wall integrity, hyphal growth, and polarity maintenance, with glucose-mediated repression of the *Afsrb1* leading to lethality [23], highlighting the critical role of protein glycosylation in fungal pathogens.

Consistent with the findings in other fungi, our data show that SsMPG2 can interact with SsMPG1 (Fig 4A and 4B), forming a complex similar to that observed in *S. pombe*. Meanwhile, the absence of *SsMPG2* affects mycelial growth and sclerotia morphology (Figs 1 and 2). It is worth noting that in the *Ssmpg2* mutants, GMPP activity was reduced, indicating that MPG2 can affect the GMPP activity of MPG1. This in turn affects the synthesis of GDP-Mannose (Fig 3) and protein glycosylation, leading to defects in cell wall integrity and virulence. Furthermore, we found that *MPG1* gene deletion is likely lethal, indicating that, as documented in *S. pombe*, *C. albicans*, and *A. fumigatus*, *MPG1* plays a crucial role in fungal growth and development [23,47]. Similar to *Ssmpg2, Ssmpg1* knockdown mutants formed deformed compound appressoria. However, they can still infect unwounded leaves, likely due to the remaining expression of *SsMPG1* in the *Ssmpg1* knockdown strains. These results suggest that the MPG1-MPG2 protein complex is maintained in eukaryotes, with MPG1 playing a major role in GDP-Mannose synthesis, while MPG2 serves a regulatory role.

Protein O-mannosylation is a conserved form of glycosylation in fungi, and defects in this process interfere with cell wall integrity and endoplasmic reticulum homeostasis [48]. O-mannosyltransferase is a key enzyme in the initiation of protein mannosylation, catalyzing the transfer of mannosyl residues from Dol-P-Man to serine and threonine residues of secreted or membrane proteins [49,50]. In this study, when *SsPMT4* was deleted in *S. sclerotiorum*, we observed reduced mycelial growth, no compound appressoria formation, and loss of pathogenicity on unwounded host tissue (Fig 5). This may be partly due to the glycosylation of Mucin Msb2, which regulates appressorium development upstream of the MAP kinase cascade [34]. Likewise, in *M*. *oryzae* and *B. cinerea*, knocking out *PMT4* homologs results in similar phenotypes, such as defective hyphal growth and cell wall integrity, and decreased virulence [35,37], indicating that the role of PMT4 in phytopathogenic fungi is also highly conserved. However, the mechanistic details of how PMT4 regulates the growth and virulence of *S. sclerotiorum* needs further exploration.

In addition to *S. sclerotiorum*, we also created gene deletions of *MPG2* in *B. cinerea*, *M*. *oryzae*, and *F. graminearum*. When *MPG2* was knocked out, the growth, infection structure, and virulence of these fungi were significantly affected (Fig 6). These results indicate that *MPG2* function is conserved across several phytopathogenic fungi, and that GDP-mannose synthesis is essential for fungal biology. Given that GDP-mannose is essential for fungal glycosylation, it may play roles in fungal cell wall modification, plant-fungal interactions, and the modification of effector proteins. However, the specific mechanisms and types of glycosylation modifications need further examination in different fungi [28].

HIGS is emerging as a powerful alternative to chemical control for protecting plants from pathogens and pests [9]. In our previous study, we identified two potent HIGS targets, *SsGAP1* and *SsSTE50*, which are involved in the growth, sclerotia formation, and virulence of *S. sclerotiorum* [13,17]. GDP-mannose is an essential precursor for protein glycosylation, and its synthesis is critical for fungal growth and virulence. Our data suggest that the components of the GDP-mannose synthesis can be used as HIGS targets for controlling *S. sclerotiorum*. When *SsMPG1* and *SsMPG2* were targeted by HIGS, enhanced resistance to *S. sclerotiorum* was observed in both *N. benthamiana* and Arabidopsis (Fig 7). Notably, the effect of *MPG1*-RNAi was more obvious than that of *MPG2*-RNAi, likely because *MPG1* plays a major role while *MPG2* plays a regulatory role in fungi. There may be more regulators within the glycosylation pathways that are essential for the virulence of *S. sclerotiorum*, that could serve as potential HIGS targets, warranting further exploration.

In summary, we characterized *SsMPG2*, which is used to stimulate GDP-mannose synthesis for protein glycosylations, which in turn affects virulence likely by altering cell wall integrity in *S. sclerotiorum*. In the future, *SsMPGs* can be used as HIGS targets to create transgenic crop plants for controlling stem rot caused by *S. sclerotiorum*.

## 4. Materials and methods

### 4.1. Fungal strains and culture conditions

*Ssoah1* in the *S. sclerotiorum* 1980 background was used as the genetic background for UV-based mutagenesis [26,27]. All knockout mutants were generated in wild-type *S. sclerotiorum* 1980 (WT) backgrounds. All strains were cultured on potato dextrose agar (PDA) (Shanghai Bio-way Technology Co., Ltd.) at room temperature. All *S. sclerotiorum* knockout mutants were screened and purified on PDA with 50 μg/ml Hygromycin B.

*B. cinerea* strains B05.10 was used as WT. All *B. cinerea* strains were cultured on PDA at room temperature. *M. oryzae* Guy11 was used as WT, and all *M. oryzae* strains were grown on complete medium (CM) in a light incubator at 25 °C with a 12h-light:12h-dark photoperiod. *F. graminearum PH-1* was used as WT. All *F. graminearum* strains were cultured on PDA at room temperature. Bacteria used in this study were grown in Luria-Bertani (LB, Sangon Biotech) medium.

### 4.2. Genomic DNA extraction and NGS analysis

DNA of all fungal sources was extracted using the CTAB method, and NGS data analysis was described previously [13]. Candidate genes were analyzed through the NCBI (National Center for Biotechnology Information) website.

### 4.3. Target gene knockout and complementation (knock-in)

The *SsMPG2*, *SsMPG1*, *SsPMT4*, *BcMPG2*, *MoMPG2*, and *FgMPG2* gene knockout (KO) cassettes were generated using overlapping PCR, and the deletion knockout mutants were obtained by homologous recombination in their corresponding protoplasts. Each KO cassette consists of target gene upstream sequences, hygromycin-resistance gene *HYG*, and target gene downstream sequences. The 7F + H855R and H855F+8R primer pair was used to identify the HYG-positive strain. The HYG-positive strains were purified multiple rounds, and PCR was performed with 5F and 6R primers to ensure the purity of the deletions.

Transgene complementation was carried out with a similar method, with the knock-in cassettes including WT *MPG2* gDNA, *HYG*, and *MPG2* downstream sequences. PCR using the 1093-F and 1093-R primer pair and Sanger sequencing were performed to ensure the fragment accuracy. All primers used for PCR are listed in S1 Table.

### 4.4. *S. sclerotiorum* growth rate determination and fungal colony morphology observation

Strains of different genotypes were grown on 90-mm diameter standard PDA plates for 3–4 days. Then, a mycelium disk from the edge of the colony was transferred with a sterilized pipette tip (4 mm diameter) to the center of a fresh PDA plate and incubated at room temperature. The colony diameter was measured every 12 hours until mycelia reached the edge of the PDA plate. Colony morphology images were taken 14 days post-inoculation for sclerotia observation.

### 4.5. Observation of infection structures

For *S. sclerotiorum* and *B. cinerea*, a fresh hyphal piece was transferred from the edge of the colony onto a glass slide using a sterilized pipette tip, and incubated for 1–2 days at room temperature on a wet paper towel inside a petri dish. The formation of compound appressoria was observed with a ZEISS light microscope.

For *M. oryzae*, conidia from the CM medium were harvested and filtered. Conidial suspension drops were inoculated on the microscope slides with coverslips and incubated at 28 °C under darkness for 12 h before observation. The formation of appressorium was observed with a ZEISS light microscope.

For *F. graminearum*, a fresh hyphal piece was transferred from the edge of the colony onto a glass slide and incubated for 2 days at room temperature on a wet paper towel inside a petri dish. The formation of infection cushions was observed with a ZEISS light microscope.

### 4.6. Plant infection assay

For *S. sclerotiorum* infection, fresh mycelial plugs (2 or 4 mm diameter) were transferred on unwounded or wounded detached *N. benthamiana* or *Arabidopsis thaliana* leaves on a wet paper towel in a petri dish. The inoculated plant leaves were incubated in a growth chamber (23 °C, 16 h light/8 h dark).

For *B. cinerea* infection, fresh mycelial plugs (2 mm in diameter) were inoculated on unwounded or wounded Arabidopsis leaves and placed on moistened paper towels in a container covered with lids to maintain humidity. Inoculated tissues were incubated under continuous darkness at 23 °C.

For *M. oryzae* infection, after the fungal strains were cultured on CM medium for 8–10 days, they were transferred to tomato oat medium for 3–4 days, and water was added to interrupt mycelial growth for spore induction. After 3 days, the spores were collected and washed, and the spore concentration was adjusted to 5x10^5^ in water. Punch inoculation of detached rice leaves was conducted with tips. A 5-μl drop of spore suspension was spotted onto each rice leaf, and the inoculated leaf was incubated in a petri dish that contained about 20ml 0.1% 6-benzylaminopurine sterile water. Lesion length was measured 7 d post-inoculation [51].

For *F. graminearum* infection, a fresh mycelial plug (2 mm diameter) of the 5-day-old culture was transferred on wheat leaves on a wet paper towel in a petri dish. The inoculated plant leaves were incubated in a growth chamber (23 °C, 16 h light/8 h dark).

The lesion sizes were quantified by ImageJ software. The virulence test was repeated twice with similar results.

### 4.7. Cell wall integrity assays

Colonies were cultured on PDA plates with cellophane for 2 days to collect mycelia. About 0.1g mycelia were collected in the tube and ground in 250 μl of 10 mM Tris, pH8 in the presence of two glass beads for four cycles of 20s each, using the TissueLyser III, QIAGEN with 20s intervals on ice. The cell suspension was collected, and the glass beads were extensively washed with cold Tris buffer for three times. The supernatant and washings were collected and centrifuged at 3800g for 5min. The pellet, containing the cell walls, was washed with cold deionized water for three times. 1ml 2N TFA (trifluoroacetic acid) was used to hydrolyze the cell wall of the hyphae, such that cell wall polysaccharides were hydrolyzed to their corresponding monomeric sugars: mannose, galactose, and glucose [52]. The suspension was heated in sealed tubes at 121°C for 1h. TFA was evaporated with N_2_ and the dry samples were re-suspended in 1 ml of MilliQ water. After centrifuging at the highest speed for 10 min, the supernatant was collected for the measurement of monomeric sugar concentration. The concentrations of the monomeric sugars were quantified by high-performance anion-exchange liquid chromatography (HPAELC) system, Dionex ICS-5000, equipped with Dionex CarboPac PA1 analytical column and guard column, as well as a pulsed amperometric detector fit with a gold electrode. The flow rate was set to 0.8 ml/min, while the mobile phase consisted Nanopure water (eluent A) from 0 to 35 min, followed by a wash phase with 0.2M NaOH (eluent B, 35 to 45 min), and then back to Nanopurewater for 15 min of equilibration. The identification of the sugars was confirmed with standards and sample spiking.

### 4.8. GMPP enzymatic activity assays

The GMPP activities in *Ssoah1*, *S1093*, WT, *Ssmpg2-1*, and *Ssmpg2-2* were measured by a commercial Microorganism GDP-mannose pyrophosphorylase (GMPase) ELISA Kit (MEI MIAN) according to the manufacturer’s instructions.

### 4.9. Glycoprotein stain

Total protein was extracted from 0.1g of fresh mycelia of 3-day-old culture. After lysis, the lysate was centrifuged at 12,000 rpm for 5 min at 4°C to collect the supernatant. 300 μl of the supernatant was mixed with 100 μl of 4× SDS loading buffer, and the mixture was heated at 95°C for 5 min. After centrifugation at 14,000 rpm for 5min, 10 μl supernatant protein was loaded and separated using SDS-PAGE with a 10% acrylamide gel. Protein glycosylation profiles were analyzed by a commercial Glycoprotein Staining Kit (Cat# MGE1924) following the manufacturer’s instructions.

### 4.10. Yeast two-hybrid (Y2H) assay

EcoRI and BamHI were used as cleavage sites for vectors pGBKT7 and pGADT7. *SsMPG2* was cloned into the vector pGBKT7 and *SsMPG1* was cloned into pGADT7 by homologous recombination. The *SsMPG2* and *SsMPG1* constructs were co-transformed into yeast strain Y2HGOLD. The colonies were grown on synthesis dropout media lacking Leu, Trp (SD-L-T) or lacking Leu, Trp, His, Ade (SD-L-T-H-A). After 3–5 days of incubation at 28 °C, the results were observed and photographed.

### 4.11. Split luciferase complementation assay

The constructs for split luciferase complementation analysis were generated as previously described [17]. DNA fragments of *MPG1* and *MPG2* were amplified from WT *S. sclerotiorum* cDNA and used to generate p35S-MPG1-nLUC and p35S-MPG2-cLUC. The constructs were introduced into Agrobacterium GV3101 and infiltrated into 4-week-old *N. benthamiana* leaves at OD_600_ = 0.5. The infiltrated leaves were treated with 1 mM luciferin after 48 hpi before luminescence detection.

### 4.12. HIGS vector construction and transient expression in *N. benthamiana*

To construct the RNAi vector of *SsMPG1*, the 422 bp sequence of the third exon of *SsMPG1* was selected as the sense strand. To construct the RNAi vector of *SsMPG2*, the 366 bp sequence of the third exon of *SsMPG2* was selected as the sense strand. Engineered sense strand, the intron 3 fragments from the malate synthase (ms-i3) gene of *A. thaliana* (will be spliced out during transcription), and the corresponding antisense strand were ligated in pCambia1300-E9 vector to create the RNAi construct as described previously [13].

The Agrobacterium GV3101 harboring empty vector (EV) pCambia1300-E9 construct or the SsMPG1RNAi and SsMPG2RNAi construct was infiltrated into 4-week-old *N. benthamiana* leaves at OD_600_ = 0.6. The plants were kept in darkness for 3 days to induce the expression of the RNAi construct. The leaves were then inoculated with *S. sclerotiorum* mycelial plugs to test for disease progression.

### 4.13. *A. thaliana* transformation of the RNAi construct

The Agrobacterium GV3101 harboring the SsMPG1RNAi or SsMPG2RNAi construct was transformed into *Arabidopsis* Col-0 plants by floral-dip protocol [53]. The transformants were selected on 1/2 MS plates with 50 μg/ml Hygromycin B. Co-segregation analysis of T2 progeny was carried out to ensure that the observed phenotype was caused by transgene expression.

## Supporting information

S1 Fig Mycelial growth rate and sequencing result of *Ss1093* and *S1093-C.* (A) Growth rate measured on PDA plates every 12 hours for 60 hours. (B) The representative DNA sequencing chromatograms for *sscle_15g102760* in *S1093* and *S1093-C.*
(TIFF)

S2 Fig Generation and analysis of *Ssmpg2* knockout alleles. (A) Targeted gene knock-out by homologous recombination. The target gene and HPH gene are shown as orange and light green rectangles, respectively. The strategy is used for the knockouts of all genes in this article. (B) PCR verification of *SsMPG2* deletion alleles. Genomic DNAs from WT *S. sclerotiorum* and two *Ssmpg2* mutants were used as PCR templates. Primer pair 1 was used to test the deletion of *SsMPG2*, and primer pairs 2 and 3 were used to test the presence of *HPH*. Lane M contains the DNA size ladder. (C) The mycelial growth rate of WT and two *Ssmpg2* mutants on PDA plates. The growth rate was measured on PDA every 12 h for 60 h.
(TIFF)

S3 Fig Phylogenetic analysis of MPG2 from different species and a standard curve for GMPP activity assay. (A) Phylogenetic analysis of MPG2 proteins from fungi, plants, and humans. The tree was built using Geneious, RaxmlGUI, and FigTree methods and evaluated by Bootstrap. The bootstrap values from 1000 replicates are labeled above the branches. The accession numbers of these proteins are AJU99767.1 (PSA1), NP_001342756.1 (MPG1), XP_663190.2(ANIA_05586), XP_958811.1 (NCU06003), APA08157.1 (SsMPG1), XP_024547842.1 (BcMPG1), XP_003714211.1 (MoMPG1), XP_011319950.1 (FgMPG1), XP_018157949.1 (CH63R_08197), NP_001189713.1 (VTC1), NP_037466.3 (GMPPB), NP_001361223.1 (GMPPA), NP_177629.1 (KJC1), NP_178542.2 (KJC2), NP_596551.1 (MPG2), XP_001554781.1 (BcMPG2), EYB30090.1 (FgMPG2), XP_018155504.1 (CH63R_08507), XP_003711770.1 `(MoMPG2), XP_001585569.1 (SsMPG2), XP_659515.2 (ANIA_01911), XP_958781.1 (NCU05937), WP_086629446.1 (ODO40_003392), PWL88500.1 (DBY14_02745). The scale bar is shown at the bottom. (B) The pyrophosphorylase consensus motifs of MPG1, MPG2, and their homologous proteins. The MPG2 family differs from the MPG1 family with two amino acid insertions, as indicated by **. (C) Standard curve for the GMPP activity assay. The X-axis indicates the concentrations of the standard used, and the Y-axis indicates the corresponding OD value. The linear regression curve of the standard was plotted, and the concentration value of each sample was calculated according to the curve equation y = 0.813x + 01758, R > 0.99.
(TIFF)

S4 Fig Generation and analysis of two independent *Ssmpg1* knock-down alleles. (A) PCR verification of the two *SsMPG1* knockdown alleles. Genomic DNAs from WT *S. sclerotiorum* and two *Ssmpg1* mutants were used as PCR templates. Primer pair 1 was used to test the deletion of *SsMPG1*, and primer pairs 2 and 3 were used to test the presence of *HPH*. Lane M contains the DNA size ladder. (B) Relative expression levels of *SsMPG1* in WT and the corresponding knock-down mutants as determined by RT-PCR. *ACTIN* was used as a control. (C) The mycelial growth rate of WT and two *Ssmpg1* mutants on PDA plates. The growth rate was measured on PDA every 12 h for 60 h. (D) Top: Virulence test of WT and two *Ssmpg1* mutants on the leaves of *N. benthamiana* at 24 hpi. Bottom: Quantification of the lesion areas caused by the indicated *S. sclerotiorum* strains. The dots represent the values of lesion areas measured by ImageJ. The experiment was repeated twice with similar results. Bar = 0.5 cm.
(TIFF)

S5 Fig Generation and analysis of two independent *Sspmt4* deletion alleles. (A) PCR verification of the *SsPMT4* knockout alleles. Genomic DNAs from WT *S. sclerotiorum* and two *Sspmt4* mutants were used as PCR templates. Primer pair 1 was used to test the deletion of *SsPMT4*, and primer pairs 2 and 3 were used to test the presence of *HPH*. Lane M contains the DNA size ladder. (B) The mycelial growth rate of WT and two *Sspmt4* mutants on PDA plates. The growth rate was measured on PDA every 12 h for 60 h. (C) Top: Virulence test of WT and the two *Sspmt4* mutants on unwounded and wounded leaves of *N. benthamiana* at 24 hpi. Bottom: Quantification of the lesion areas caused by the indicated *S. sclerotiorum* strains. The dots represent the values of lesion areas measured by ImageJ. The experiment was repeated twice with similar results. Bar = 0.5 cm. All statistical analyses were carried out by Student’s *t*-test.
(TIFF)

S6 Fig Generation of independent *MPG2* deletion alleles by homologous recombination in *B. cinerea*, *M. oryzae*, and *F. graminearum.* (A) PCR verification of the *BcMPG2* deletion alleles. Genomic DNAs from WT *B. cinerea* B05.10 and two *Bcmpg2* mutants were used as PCR templates. Primer pair 1 was used to test the deletion of *BcMPG2*, and primer pairs 2 and 3 were used to test the presence of *HPH*. Lane M contains the DNA size ladder. (B) PCR verification of the *MoMPG2* deletion alleles. Genomic DNAs from WT *M. oryzae* Guy11 and two *Mompg2* mutants were used as PCR templates. Primer pair 1 was used to test the deletion of *MoMPG2* and primer pairs 2 and 3 were used to test the presence of *HPH*. Lane M contains the DNA size ladder. (C) PCR verification of the *FgMPG2* deletion alleles. Genomic DNAs from WT *F. graminearum PH-1* and two *Fgmpg2* mutants were used as PCR templates. Primer pair 1 was used to test the deletion of *FgMPG2* and primer pairs 2 and 3 were used to test the presence of *HPH*. Lane M contains the DNA size ladder.
(TIFF)

S7 Fig Schematic representation of *SsMPG1 and SsMPG2* RNAi constructs pC1300-*SsMPG1*RNAi-E9 and pC1300-*SsMPG2*RNAi-E9 for dsRNA generation after expression in the plant hosts. (TIFF)

S1 Table Primers used for this study. (XLSX)

S2 Table Source data. (XLSX)
