# Supplementary figures and images for: A GDP-mannose-1-phosphate guanylyltransferase as a potential HIGS target against Sclerotinia sclerotiorum
Source: PLoS Pathog. 2025 May 2;21(5):e1013129. doi: 10.1371/journal.ppat.1013129 (PMC12068732; doi:10.1371/journal.ppat.1013129)

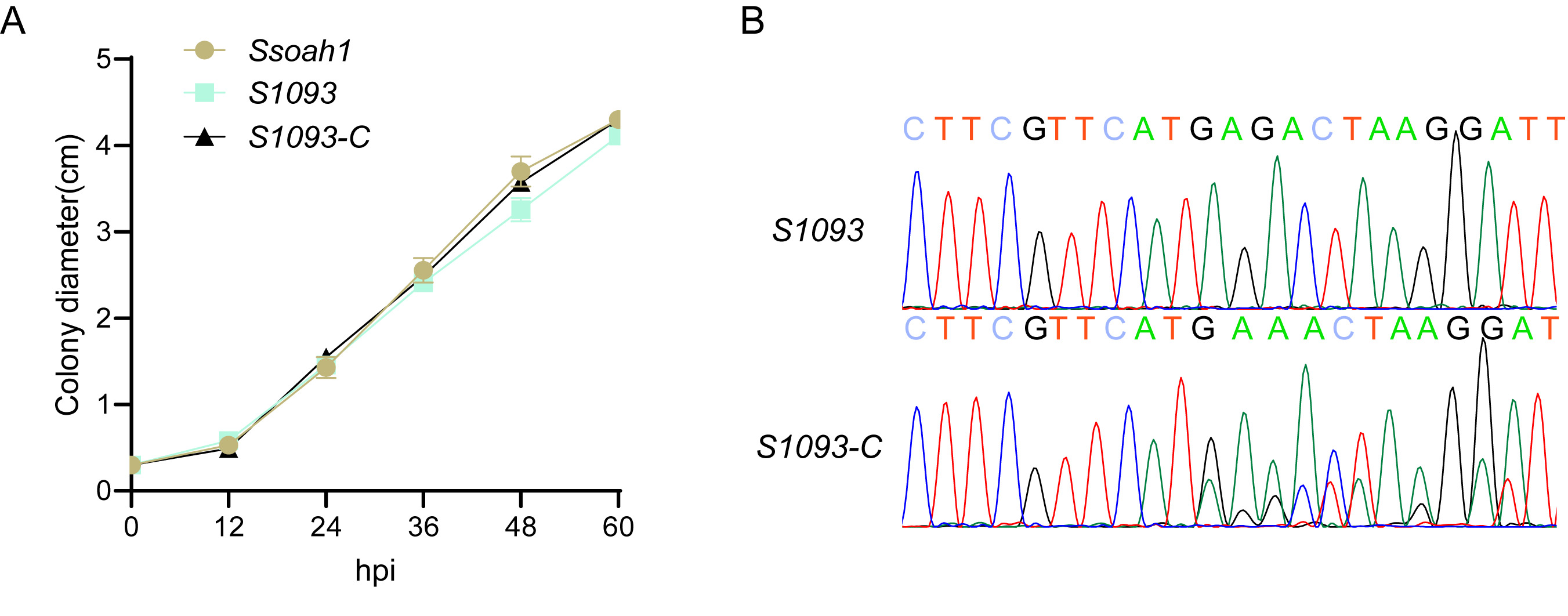

Supplement: S1 Fig — (A) Growth rate measured on PDA plates every 12 hours for 60 hours. (B) The representative DNA sequencing chromatograms for sscle_15g102760 in S1093 and S1093-C. (TIFF) [file ppat.1013129.s001.tiff]

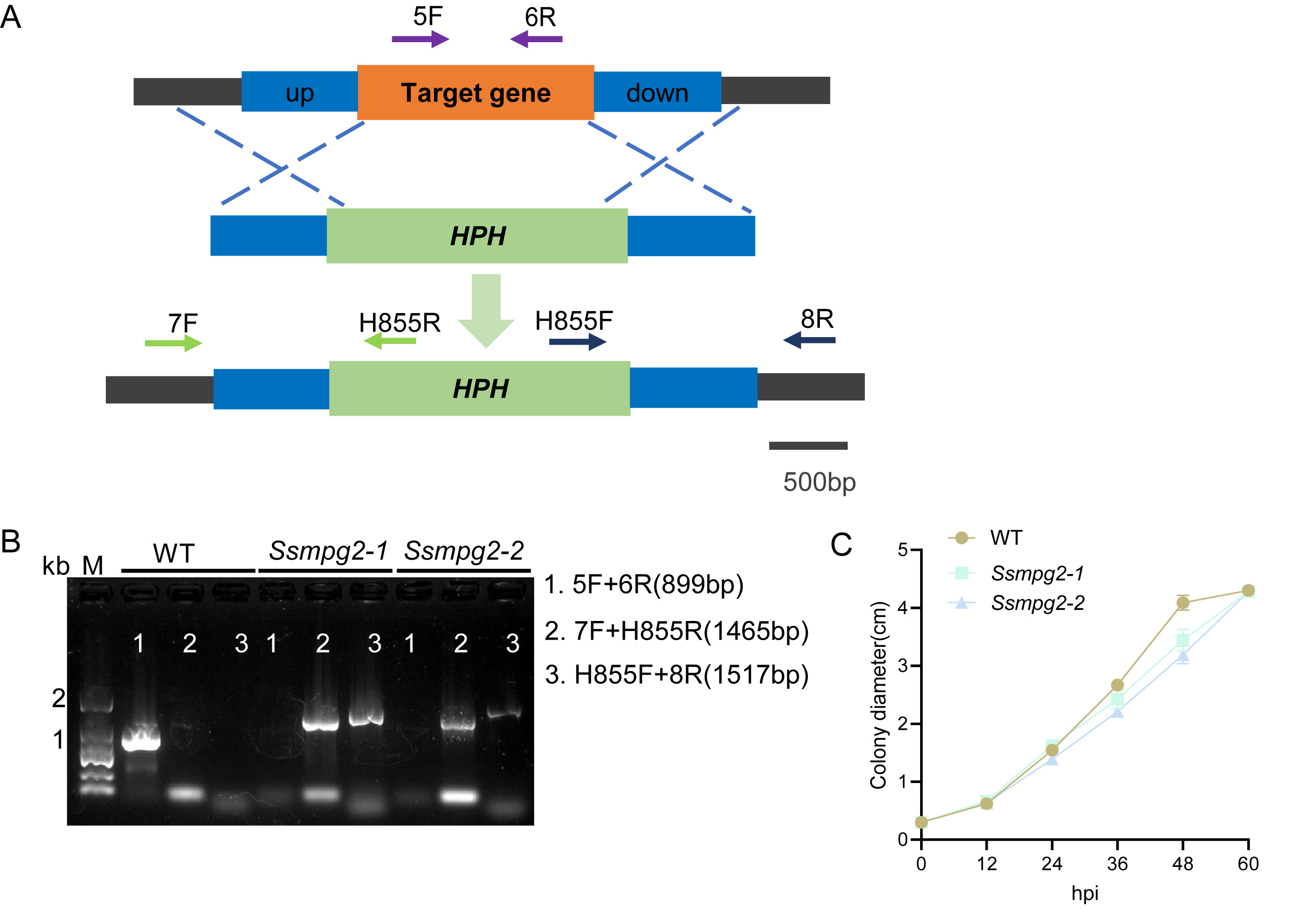

Supplement: S2 Fig — (A) Targeted gene knock-out by homologous recombination. The target gene and HPH gene are shown as orange and light green rectangles, respectively. The strategy is used for the knockouts of all genes in this article. (B) PCR verification of SsMPG2 deletion alleles. Genomic DNAs from WT S. sclerotiorum and two Ssmpg2 mutants were used as PCR templates. Primer pair 1 was used to test the deletion of SsMPG2, and primer pairs 2 and 3 were used to test the presence of HPH. Lane M contains the DNA size ladder. (C) The mycelial growth rate of WT and two Ssmpg2 mutants on PDA plates. The growth rate was measured on PDA every 12 h for 60 h. (TIFF) [file ppat.1013129.s002.tiff]

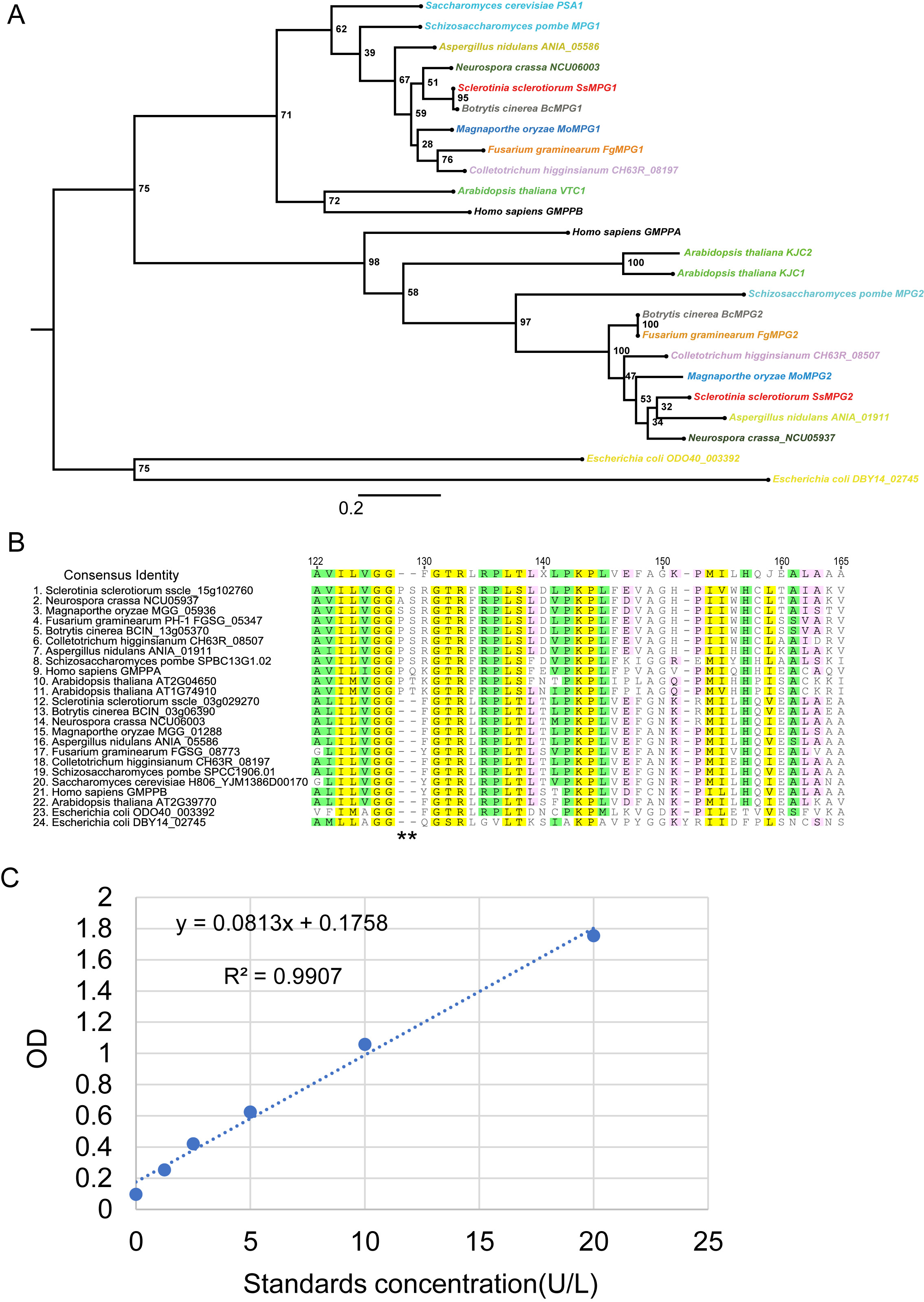

Supplement: S3 Fig — (A) Phylogenetic analysis of MPG2 proteins from fungi, plants, and humans. The tree was built using Geneious, RaxmlGUI, and FigTree methods and evaluated by Bootstrap. The bootstrap values from 1000 replicates are labeled above the branches. The accession numbers of these proteins are AJU99767.1 (PSA1), NP_001342756.1 (MPG1), XP_663190.2(ANIA_05586), XP_958811.1 (NCU06003), APA08157.1 (SsMPG1), XP_024547842.1 (BcMPG1), XP_003714211.1 (MoMPG1), XP_011319950.1 (FgMPG1), XP_018157949.1 (CH63R_08197), NP_001189713.1 (VTC1), NP_037466.3 (GMPPB), NP_001361223.1 (GMPPA), NP_177629.1 (KJC1), NP_178542.2 (KJC2), NP_596551.1 (MPG2), XP_001554781.1 (BcMPG2), EYB30090.1 (FgMPG2), XP_018155504.1 (CH63R_08507), XP_003711770.1 `(MoMPG2), XP_001585569.1 (SsMPG2), XP_659515.2 (ANIA_01911), XP_958781.1 (NCU05937), WP_086629446.1 (ODO40_003392), PWL88500.1 (DBY14_02745). The scale bar is shown at the bottom. (B) The pyrophosphorylase consensus motifs of MPG1, MPG2, and their homologous proteins. The MPG2 family differs from the MPG1 family with two amino acid insertions, as indicated by **. (C) Standard curve for the GMPP activity assay. The X-axis indicates the concentrations of the standard used, and the Y-axis indicates the corresponding OD value. The linear regression curve of the standard was plotted, and the concentration value of each sample was calculated according to the curve equation y = 0.813x + 01758, R > 0.99. (TIFF) [file ppat.1013129.s003.tiff]

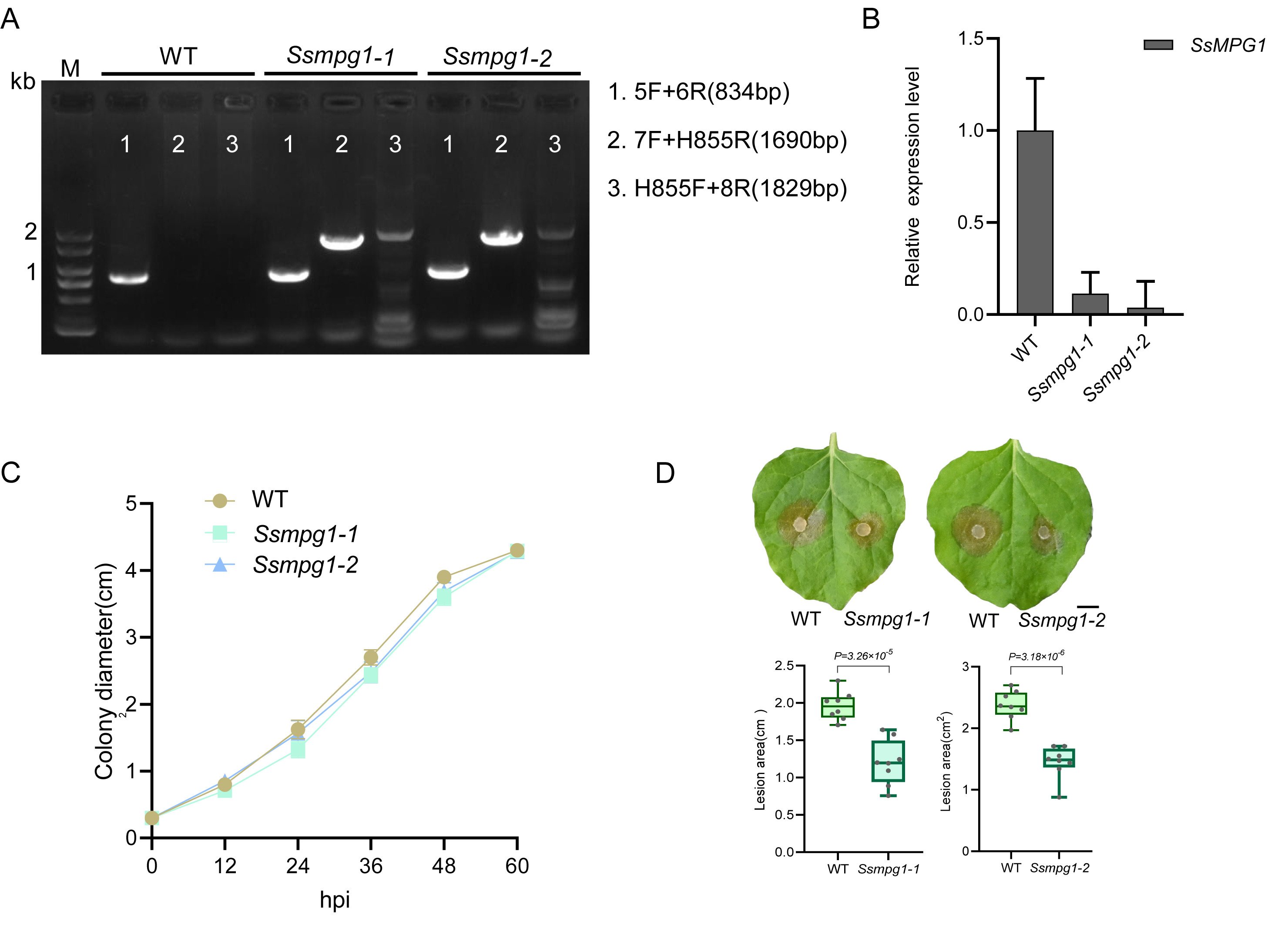

Supplement: S4 Fig — (A) PCR verification of the two SsMPG1 knockdown alleles. Genomic DNAs from WT S. sclerotiorum and two Ssmpg1 mutants were used as PCR templates. Primer pair 1 was used to test the deletion of SsMPG1, and primer pairs 2 and 3 were used to test the presence of HPH. Lane M contains the DNA size ladder. (B) Relative expression levels of SsMPG1 in WT and the corresponding knock-down mutants as determined by RT-PCR. ACTIN was used as a control. (C) The mycelial growth rate of WT and two Ssmpg1 mutants on PDA plates. The growth rate was measured on PDA every 12 h for 60 h. (D) Top: Virulence test of WT and two Ssmpg1 mutants on the leaves of N. benthamiana at 24 hpi. Bottom: Quantification of the lesion areas caused by the indicated S. sclerotiorum strains. The dots represent the values of lesion areas measured by ImageJ. The experiment was repeated twice with similar results. Bar = 0.5 cm. (TIFF) [file ppat.1013129.s004.tiff]

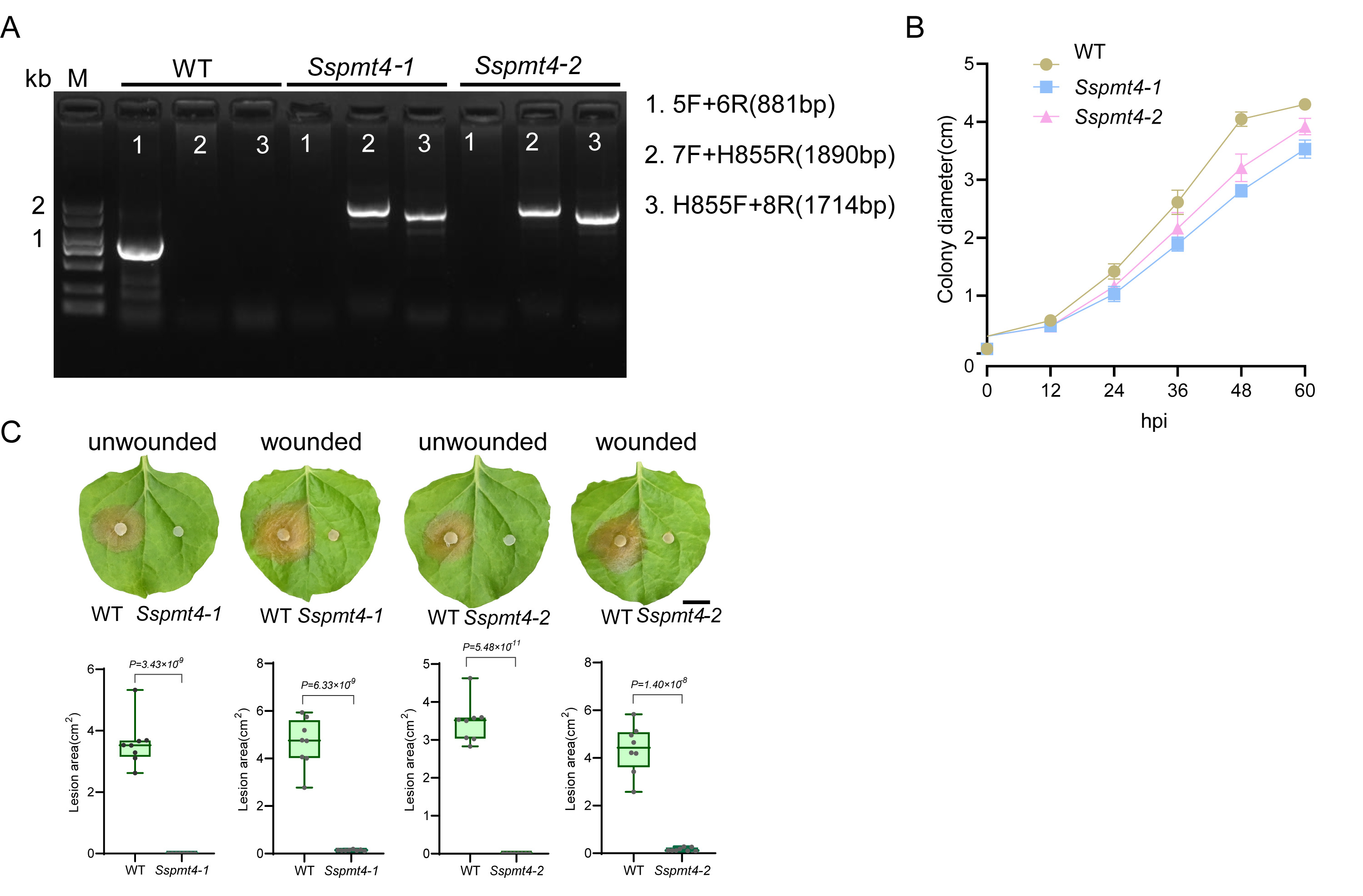

Supplement: S5 Fig — (A) PCR verification of the SsPMT4 knockout alleles. Genomic DNAs from WT S. sclerotiorum and two Sspmt4 mutants were used as PCR templates. Primer pair 1 was used to test the deletion of SsPMT4, and primer pairs 2 and 3 were used to test the presence of HPH. Lane M contains the DNA size ladder. (B) The mycelial growth rate of WT and two Sspmt4 mutants on PDA plates. The growth rate was measured on PDA every 12 h for 60 h. (C) Top: Virulence test of WT and the two Sspmt4 mutants on unwounded and wounded leaves of N. benthamiana at 24 hpi. Bottom: Quantification of the lesion areas caused by the indicated S. sclerotiorum strains. The dots represent the values of lesion areas measured by ImageJ. The experiment was repeated twice with similar results. Bar = 0.5 cm. All statistical analyses were carried out by Student’s t-test. (TIFF) [file ppat.1013129.s005.tiff]

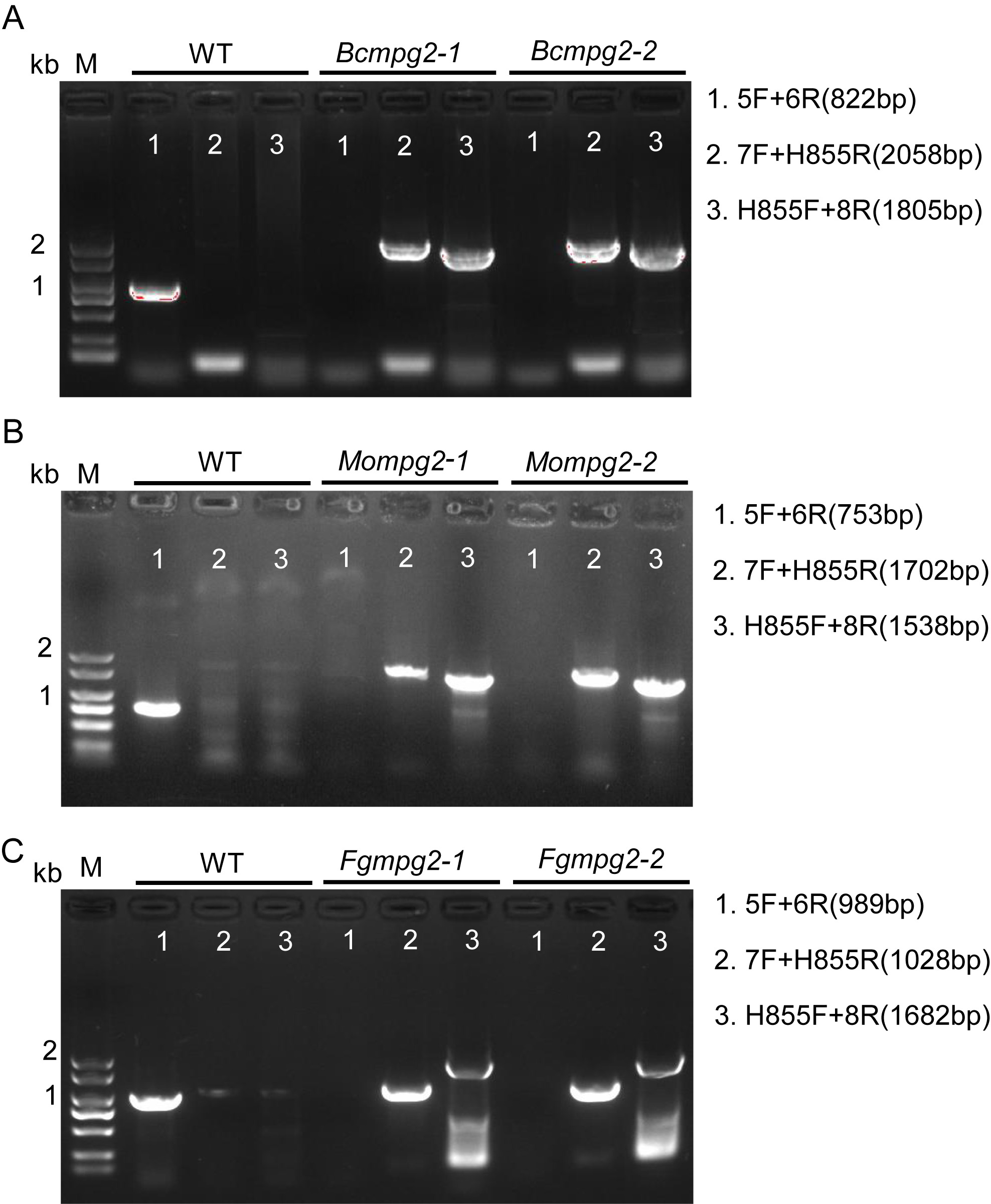

Supplement: S6 Fig — (A) PCR verification of the BcMPG2 deletion alleles. Genomic DNAs from WT B. cinerea B05.10 and two Bcmpg2 mutants were used as PCR templates. Primer pair 1 was used to test the deletion of BcMPG2, and primer pairs 2 and 3 were used to test the presence of HPH. Lane M contains the DNA size ladder. (B) PCR verification of the MoMPG2 deletion alleles. Genomic DNAs from WT M. oryzae Guy11 and two Mompg2 mutants were used as PCR templates. Primer pair 1 was used to test the deletion of MoMPG2 and primer pairs 2 and 3 were used to test the presence of HPH. Lane M contains the DNA size ladder. (C) PCR verification of the FgMPG2 deletion alleles. Genomic DNAs from WT F. graminearum PH-1 and two Fgmpg2 mutants were used as PCR templates. Primer pair 1 was used to test the deletion of FgMPG2 and primer pairs 2 and 3 were used to test the presence of HPH. Lane M contains the DNA size ladder. (TIFF) [file ppat.1013129.s006.tiff]

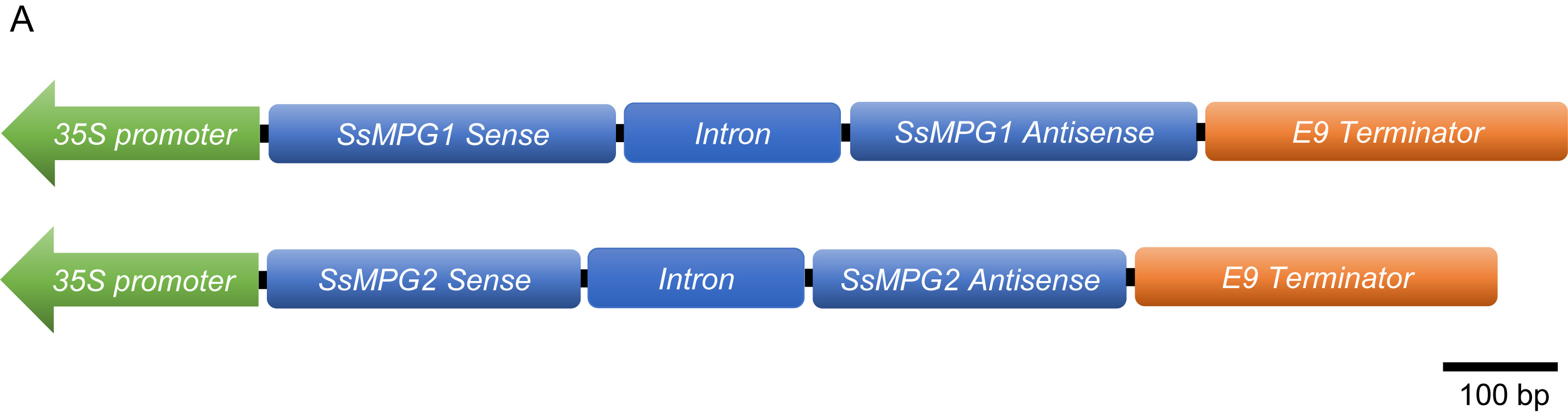

Supplement: S7 Fig — (TIFF) [file ppat.1013129.s007.tiff]
